# Supplementary material for: Impact of the Genotype and Phenotype of CYP3A and P-gp on the Apixaban and Rivaroxaban Exposure in a Real-World Setting
Source: J Pers Med. 2022 Mar 24;12(4):526. doi: 10.3390/jpm12040526 (PMC9028714; doi:10.3390/jpm12040526)
Supplement: Supplementary file 1 [file jpm-12-00526-s001.zip › jpm-1629834-supplementary.pdf]

Journal of Personalized Medicine

MDPI

Supplementary Materials

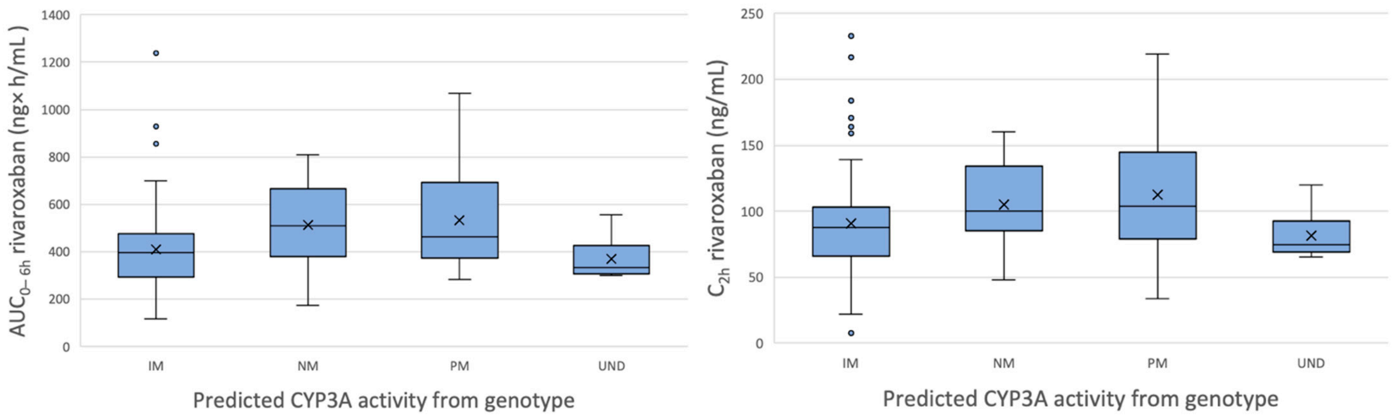

(**a**) (**b**)

Figure S1. (**a**) AUC_{0-6h} and (**b**) C_{2h} of rivaroxaban according to the CYP3A predicted activity from genotype.

Table S1. SNPs of *CYP3A4* and *CYP3A5* studied.

Gene	rs Number	Common Allele Name
CYP3A4	rs12721629	CYP3A4*12,c.1117 C>T,g.21896C>T
	rs4987161	CYP3A4*17,c.566 T>C,g.15615T>C
	rs2740574	CYP3A4*1B, g.-392A>G
	rs55785340	CYP3A4*2,c.664T>C,g.15713T>C
	rs35599367	CYP3A4*22, g.15389C>T
	rs4986910	CYP3A4*3,c.1334T>C
CYP3A5	rs28365083	CYP3A5*2,g.27289C>A
	rs776746	CYP3A5*3/*10,g.6986A>G
	rs28383468	CYP3A5*3B,g.3705C>T
	rs10264272	CYP3A5*6,g.14690G>A
	rs41303343	CYP3A5*7,g.27131_27132insT
	rs55817950	CYP3A5*8,g.3699C>T
	rs28383479	CYP3A5*9,g.19386G>A

Table S2. Frequencies of the different genotypes found in our cohort as compared to frequencies found in reference population (Caucasian).

rs ID	Homozygous for Major Allele (Cohort)	Homozygous for Major Allele (Reference)	Heterozygous (Cohort)	Heterozygous (Reference)	Homozygous for Minor Allele (cohort)	Homozygous for Minor Allele (Reference)
rs10264272	0.993	0.994	0.007	0.006	0.000	0.000
rs12721629	1.000	1.000	0.000	0.000	0.000	0.000
rs2740574	0.936	0.946	0.064	0.052	0.000	0.002
rs28365083	0.993	0.992	0.007	0.008	0.000	0.000
rs28383468	0.969	0.978	0.031	0.020	0.000	0.002
rs28383479	1.000	1.000	0.000	0.000	0.000	0.000
rs35599367	0.892	0.903	0.105	0.095	0.003	0.002
rs41303343	1.000	1.000	0.000	0.000	0.000	0.000
rs4986910	0.980	0.986	0.017	0.014	0.003	0.000
rs4987161	1.000	1.000	0.000	0.000	0.000	0.000
rs55785340	0.997	0.996	0.000	0.0004	0.003	0.000
rs55817950	1.000	1.000	0.000	0.000	0.000	0.000
rs776746	0.881	0.891	0.112	0.105	0.007	0.004
rs1045642	0.264	0.266	0.458	0.503	0.278	0.231
rs2032582	0.311	0.316	0.451	0.489	0.239	0.159
rs1128503	0.315	0.334	0.468	0.501	0.217	0.165

Table S3. Multivariable linear regression models to assess if the phenotypic activity of CYP3A and P-gp are associated with the C_{2h} of apixaban and rivaroxaban. Each independent variable is reported with its beta coefficient (β) and its 95% confidence interval (CI95%).

	**C_{2h} of Apixaban**	**C_{2h} of Rivaroxaban**
	R^2	
	46%	22%
Intercept	−0.46 (−51.98 to 51.05); *p* = 0.9859	−77.74 (−161.1 to 5.63); *p* = 0.0673
Variables		
$MR_{midazolam}$, per log_{10}	6.07 (−8.08 to 20.22); *p* = 0.3979	−8.35 (−30.17 to 13.47); *p* = 0.4503
$AUC_{fexofenadine}$, per log_{10}	31.52 (14.92 to 48.13); *p* = 0.0003	47.19 (19.46 to 74.93); *p* = 0.001
Weight, per kg	−0.02 (−0.37 to 0.33); *p* = 0.9097	0.23 (−0.18 to 0.63); *p* = 0.2713
CrCl, per unit	−0.40 (−0.67 to −0.13); *p* = 0.0042	−0.017(−0.49 to 0.34); *p* = 0.7212
ALAT, per unit	0.06 (−0.13 to 0.25); *p* = 0.5397	0.05 (−0.14 to 0.23); *p* = 0.6233
Gender		
Male	Reference category	Reference category
Female	4.07 (−5.94 to 14.08); *p* = 0.4233	1.91 (−13.03 to 16.84); *p* = 0.8009
Dose		
2.5 mg bid	Reference category; *p* < 0.0001*	NA
5 mg bid	51.97 (41.16 to 62.78); *p* < 0.0001	NA
10 mg bid	116.28 (80.44 to 152.12); *p* < 0.0001	NA
10 mg od	NA	Reference category; *p* = 0.0140*
15 mg od	NA	18.95 (−21.77 to 59.66); *p* = 0.3587
20 mg od	NA	39.98 (−4.47 to 84.44); *p* = 0.0775
15 mg bid	NA	41.5 (0.24 to 82.75); *p* = 0.0487
Age		
<65 years	Reference category; *p* = 0.4188*	Reference category; *p* = 0.2714*
65–74 years	8.61 (−10.00 to 27.23); *p* = 0.3619	15.15 (−0.06 to 30.37); *p* = 0.051
75–84 years	13.69 (−5.59 to 32.97); *p* = 0.1627	10.55 (−12.65 to 33.75); *p* = 0.3696
>85 years	17.73 (−3.37 to 38.82); *p* = 0.0990	7.97 (−15.72 to 31.67); *p* = 0.5066

**p*-value for the overall association between C_{2h} and the variable. Abbreviations: CrCl, creatinine clearance; ALAT, alanine transaminase; MR, metabolic ratio; AUC, area under the curve; C_{2h}, concentration 2 h after drug administration; bid, twice daily; od, once daily; NA, not applicable.

Table S4. Multivariable linear regression models to assess if the genotype of CYP3A and P-gp are associated with the C_{2h} of apixaban and rivaroxaban. Each independent variable is reported with its beta coefficient (β) and its 95% confidence interval (CI95%).

	**C_{2h} of Apixaban**	**C_{2h} of Rivaroxaban**
	R^2	
	39%	15%
Intercept	80.68 (42.43 to 118.93); $p = 0.0001$	−10.99 (−88.50 to 66.51); $p = 0.7792$
Variables		
Weight, per kg	0.01 (−0.38 to 0.36); $p = 0.9693$	0.46 (0.04 to 0.88); **$p = 0.0328$**
CrCl, per unit	−0.48 (−0.75 to −0.21); **$p = 0.0006$**	−0.14 (−0.61 to 0.32); $p = 0.5481$
ALAT, per unit	0.07 (−0.17 to 0.32); $p = 0.5684$	0.08 (−0.06 to 0.22); $p = 0.2719$
Predicted phenotype from genotype CYP3A		
IM	Reference category; $p = 0.1103$*	Reference category; **$p = 0.0482$***
NM	−2.16 (−23.45 to 19.13); $p = 0.8413$	20.74 (1.20 to 40.28); $p = 0.0378$
PM	−18.61 (−36.00 to −1.21); $p = 0.0362$	24.72 (−8.3 to 57.73); $p = 0.1407$
Genotype *ABCB1* 1236C>T		
No mutation	Reference category; $p = 0.7096$*	Reference category; $p = 0.4203$*
Heterozygous for mutation	−1.26 (−23.09 to 20.57); $p = 0.9093$	−16.43 (−46.63 to 13.77); $p = 0.2834$
Homozygous for mutation	−9.35 (−36.76 to 18.06); $p = 0.5013$	−1.63 (−39.30 to 36.03); $p = 0.9316$
Genotype *ABCB1* 3435C>T		
No mutation	Reference category; $p = 0.6778$*	Reference category; $p = 0.4836$*
Heterozygous for mutation	−7.52 (−24.64 to 9.60); $p = 0.3866$	−8.53 (−32.11 to 15.04); $p = 0.4747$
Homozygous for mutation	−2.70 (−18.04 to 12.65); $p = 0.7288$	−12.17 (−32.48 to 8.14); $p = 0.2375$
Genotype *ABCB1* 2677G>T		
No mutation	Reference category; $p = 0.7470$*	Reference category; $p = 0.3409$*
Heterozygous for mutation	8.08 (−14.86 to 31.01); $p = 0.4874$	23.36 (−8.85 to 55.56); $p = 0.1535$
Homozygous for mutation	9.94 (−17.81 to 37.69); $p = 0.4800$	16.54 (−19.70 to 52.77); $p = 0.3677$
Gender		
Male	Reference category	Reference category
Female	5.44 (−5.98 to 16.87); $p = 0.3206$	2.56 (−12.47 to 17.59); $p = 0.7361$
Dose		
2.5 mg bid	Reference category; **$p < 0.0001$***	NA
5 mg bid	51.17 (37.82 to 64.51); $p < 0.0001$	NA

10 mg bid	113.17 (81.73 to 145.52); $p < 0.0001$	NA
10 mg od	NA	Reference category; $\mathbf{p = 0.0002}$*****
15 mg od	NA	29.53 (−2.56 to 61.63); $p = 0.0709$
20 mg od	NA	57.40 (22.52 to 92.28); $p = 0.0015$
15 mg bid	NA	60.99 (29.95 to 92.04); $p = 0.0002$
Age		
<65 years	Reference category; $p = 0.7224$*	Reference category; $\mathbf{p = 0.0284}$*****
65–74 years	5.10 (−15.52 to 25.71); $p = 0.6257$	24.56 (8.41 to 40.71); $p = 0.0032$
75–84 years	9.29 (−10.54 to 29.12); $p = 0.3560$	25.66 (−1.56 to 52.88); $p = 0.0644$
>85 years	14.31 (−10.69 to 39.31); $p = 0.2597$	19.86 (−9.35 to 49.08); $p = 0.1806$

*p-value for the overall association between C_{2h} and the variable. Abbreviations: CrCl, creatinine clearance; ALAT, alanine transaminase; IM, intermediate metabolizer; NM, normal metabolizer, PM, poor metabolizer; bid, twice daily; od, once daily; NA, not applicable. Statistically significant values are marked with bold.

Table S5. Spearman's correlation between phenotype activity of CYP3A activity predicted by genotype and $MR_{midazolam}$ and between genotypes of P-gp and $AUC_{fexofenadine}$ for apixaban and rivaroxaban cohorts.

	Apixaban	Rivaroxaban
CYP3A activity predicted by genotype and $MR_{midazolam}$	$\varrho = 0.123$; ($p = 0.121$)	$\varrho = 0.163$; ($p = 0.065$)
Genotype of *ABCB1* 1236C>T and $AUC_{fexofenadine}$	$\varrho = -0.050$; ($p = 0.530$)	$\varrho = -0.060$; ($p = 0.496$)
Genotype of *ABCB1* 2677G>T and $AUC_{fexofenadine}$	$\varrho = -0.011$; ($p = 0.887$)	$\varrho = 0.026$; ($p = 0.772$)
Genotype of *ABCB1* 3435C>T and $AUC_{fexofenadine}$	$\varrho = 0.013$; ($p = 0.870$)	$\varrho = -0.056$; ($p = 0.528$)
